# How to reduce the adverse effects of weight stigma on the quality of life: a preferred reported items for systematic reviews and meta-analyses (PRISMA)

**DOI:** 10.3389/fpsyg.2024.1421609

**Published:** 2024-12-24

**Authors:** Guillaume Ramsamy, Helena Mosbah, Jean Pierre Faure, Vanina Plault, Marion Albouy, Catherine Esnard

**Affiliations:** ^1^Département de psychologie, Université de Poitiers, Université François Rabelais de Tours, CNRS, Poitiers, UMR7295 Centre de recherches sur la cognition et l’apprentissage (CeRCA), Poitiers, France; ^2^Centre spécialisé de l’obésité, Centre Hospitalier Universitaire (CHU) de Poitiers, Poitiers, France; ^3^Department of Psychology and the Department of Medicine, UMR7267 Ecologie et biologie des interactions (EBI), Poitiers, France

**Keywords:** weight stigma, weight bias internalization, obesity, quality of life, intervention, health

## Abstract

**Introduction:**

Obesity, affecting 38% of adults globally, carries economic burdens and health risks like cardiovascular disease and diabetes. Weight-loss programs often face challenges due to stigma and poor body image, impacting individuals’ quality of life. Research on interventions targeting weight stigma is lacking, emphasizing the need for comprehensive approaches addressing psychological and behavioral aspects for effective care.

**Methods:**

A systematic literature review was conducted according to PRISMA guidelines. We searched into three databases (PubMed, APA PsycArticles/PsycInfo, and Web of Science) articles published between 1975 and 2024. Studies were eligible if they involved people living with overweight or obesity who participate in a psychological program targeting, or not, weight stigma and if at least one outcome was related to weight stigma.

**Results:**

We selected 24 studies published between 2009 and 2022, the majority concerning English-speaking countries. Reduction in weight stigma was observed in 23/24 studies, particularly through cognitive-behavioral techniques (18/24 studies), while others studies emphasized individual predispositions and the need for longer, and denser interventions.

**Conclusion:**

Three relevant characteristics emerged from the studies analysed: content, duration and tools. Cognitive-behavioral techniques were central, aiding participants in managing their condition and coping with stigma. Interventions reducing Weight Bias Internalization (WBI) led to improved psychosocial determinants, yet the mechanisms remain unclear. Future research should address intervention duration, participant involvement, and the association between WBI and psychosocial factors to enhance outcomes and understanding.

## Introduction

Obesity is a chronic, progressive condition characterized by a body mass index (BMI) over 30 kg/m^2^ and defined by an abnormal or excessive accumulation of body fat (Bray et al., 2017). Affecting approximately 38% of the world’s adult population in 2020, obesity is projected to concern over half the global population (51%) by 2035 (World Obesity Federation, 2023). Beyond its economic impact—estimated at nearly US$2 billion in 2020 and set to the double by 2035—obesity significantly compromises individual health and productivity. It is a risk factor for chronic conditions, including cardiovascular disease, type 2 diabetes, and certain cancers (The GBD 2015 Obesity Collaborators, 2017). Thus, obesity is a major public health issue. Traditional obesity treatments focus on significant weight loss through intensive behavioral intervention targeting diet and physical activity (Lasikiewicz et al., 2014). While these programs can yield short-term benefits, they are often unsustainable, with patients dropping out of treatment and regaining weight within the first years post-treatment (Jensen et al., 2014; Wadden et al., 2012). Increasing awareness of obesity’s etiology has highlighted the role of psychosocial factors in weight management, such as body image, self-esteem, stress (MacLean et al., 2018; Wing and Phelan, 2005). For instance, poor body image and negative talk self-talk can hinder weight loss efforts (Striegel-Moore et al., 2001). Among the psychosocial factors, weight stigma has emerged as a critical but underexplored dimension influencing both psychological and physical health outcomes.

Weight stigma refers to negative societal attitudes and stereotypes about individuals with obesity (Pont et al., 2017; Puhl and Brownell, 2001; Puhl, 2011; Puhl and Heuer, 2009), such as being lazy and lacking willpower (Pearl, 2018). Such stigmatization contributes to a harmful stress, overeating, and shame, as described in cyclic obesity/weight-based stigma (COBWEBS) model (Tomiyama, 2014). Chronic exposure to stigmatizing environments triggers psychological and behavioral responses, including heightened cortisol levels, disordered eating behaviors, and reduced motivation for salutogenic behaviors (Dickerson and Kemeny, 2004; Muennig, 2008; Puhl and Brownell, 2006). Over time individuals internalize these stereotypes, applying to themselves, which exacerbates emotional distress. Such experiences trigger stereotype threat effect (Steele and Aronson, 1995), creating fear of confirming weight-based stereotypes and hindering salutogenic behaviors like physical activity and dietary changes (Brochu and Dovidio, 2014; Schvey et al., 2011; Seacat and Mickelson, 2009). This internalization is a key factor affecting mental and physical health (Pearl and Puhl, 2018).

Despite its widespread impact, weight stigma remains under-addressed in obesity management programs. Furthermore, to our knowledge, there is a lack of comprehensive reviews evaluating intervention specifically designed to reduce weight stigma and improve quality of life for individuals with obesity. This present review aims to fill this gap by examining the psychological (e.g., self-esteem, body image) and behavioral (e.g., salutogenic practices) benefits of interventions targeting weight stigma, but also to provide an update on current methodologies and strategies for addressing weight stigma. By comparing and evaluating different intervention approaches, this paper seeks to contribute to the development of holistic, stigma-sensitive approaches to obesity care, ultimately improving the quality of life and health outcomes for individuals living with obesity.

## Materials and methods

### Information sources and research process

In order to identify these psychological and behavioral benefits, we conducted a literature review by searching three academic databases (PubMed, APA PsycArticles/PsycInfo, and Web of Science) for articles published between 1975 and January 2024. Search terms covered the following three topics: (1) stigmatization (psychosocial terms), (2) obesity (population terms), and (3) therapy (intervention terms).

The keyword search was based on the MEDLINE algorithm. We defined the following list of keywords: (weight stigma or prejudice or social discrimination) AND (obesity or overweight or obese) AND (treatment or intervention or therapy). Then we applied this algorithm to PubMed, APA PsycArticles/PsycInfo, and Web of Science. We also conducted free searches consisting of additional manual searches of the first pages of results from the generic web search engine Google Scholar. We included articles considered to be of interest by examining references cited in the included studies. Then we merged these searches and included them in a library we created in Zotero 5.0.95.1. We screened eligible criteria (inclusion and exclusion), read titles and abstracts in order to deleted duplicates. We assessed whether all inclusion criteria had been met and whether the abstracts presented any exclusion criteria. Abstracts that did not meet all these criteria were excluded from the final selection of articles.

### Research question and scope

To structure the research question comprehensively, PICOS framework was applied. The population focused on adults experiencing weight stigma in social or healthcare settings. Interventions included psychological and behavioral programs aimed at addressing weight stigma or its psychological effects. Comparators included standard care, alternative psychological interventions, or no treatment, allowing for evaluation of intervention-specific effects. Outcomes assessed included reductions in weight stigma, improvement in quality of life and body image, and secondary effects such as adoption of salutogenic behaviors and reduced depressive symptoms. Study designs ranged from randomized controlled trials to quasi-experimental and observational studies, incorporating both quantitative and qualitative methods for a comprehensive analysis.

### Eligibility criteria and study selection

The inclusion criteria were as follows:

- studies in which outcomes (e.g., psychosocial determinant, self-efficacy, weight stigma) were clearly defined
- studies in which the independent variable (i.e., populations recognized as targets of weight stigmatization) was present in the context of an intervention program
- studies in which the population was defined by the authors as living with overweight or obesity

The exclusion criteria were as follows:

- studies not written in English
- studies that are not journal articles (e.g., books, chapters, editorial reviews, literature reviews, etc.)
- studies in which the dependent variable did not concern quality of life, well-being, or weight stigma
- studies without an interventional design testing the reduction of stigmatization’s adverse effects
- studies outside the context of a psychological intervention (e.g., medication, law, etc.)
- studies of populations not defined by the authors as living with overweight or obesity
- studies concerning the evaluation of a measurement tool (e.g., validation of a test or questionnaire).

### Data collection process

The review was conducted in accordance with the Preferred Reporting Items for Systematic Reviews and Meta Analyses (PRISMA) statement (Page et al., 2021), which ensures the transparent and complete reporting of systematic reviews. The articles were selected by the first author (GR), and this selection was then checked independently by three coauthors (CE, MA, VP). Any uncertainty or disagreement between evaluators was resolved by discussion and consensus among the authors (GR, VP, MA, CE). The first author (GR) extracted the following information: first authors, year of publication, journal, study country, study population, study population size, method, data collection, dependent variables, and summary of results.

### Assessing the risk of bias

The first author (GR) independently assessed the methodological quality of the studies included using the modified version of the Downs and Black checklist for assessing randomized controlled trials and non-randomized controlled trials (Downs and Black, 1998). Downs and Black score ranges were given to corresponding quality levels as previously reported (Hooper et al., 2008): excellent (26–28); good (20–25); fair (15–19); and poor (< 14).

## Results

### Selection of the studies

**Figure 1:**
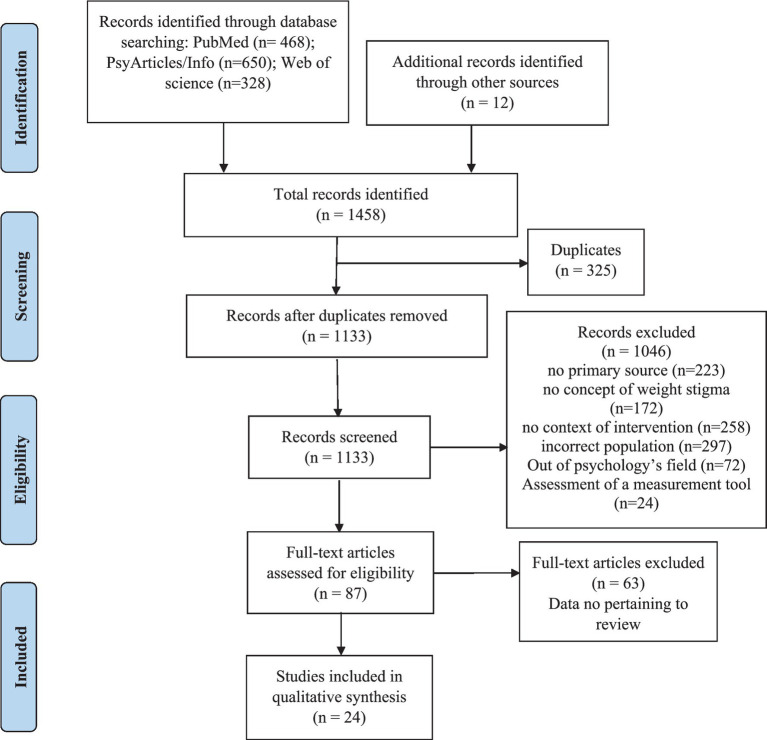
PRISMA flow diagram.

The study procedure is illustrated in Figure 1. The free search yielded 12 articles. Keyword searching with the MEDLINE algorithm yielded 468 results in PubMed, 650 results in APA PsychArticles/Info, and 328 results in Web of Science. After removing duplicates (325), 1,046 articles were removed based on the title or abstract because they did not meet criteria for inclusion. Then, after reading and analyzing the 87 articles, we (GR, VP, CE, MA) included 24 articles based on the eligibility criteria.

### Characteristics of the studies

The characteristics of the studies are set out in Table 1. All studies selected were published between 2009 and 2022. Twenty articles concerned English-speaking countries (92% of which were from the United States), three articles concerned Europe, and one article concerned Brazil. All studies included a population living with overweight or obesity. The mean BMI was always over 30 kg/m^2^, with the exception of the study by Wallin et al. (2018), whose mean BMI was 29.9 kg/m^2^. Participants were predominantly women over the age of 18. Quality of life dimensions assessed overall obesity-related quality of life, physical, and psychological factors.

**Table 1:** Characteristics of studies included in review.

Authors	Journal	Study year	Country	Sample size	Mean BMI in Kg/m^2^	Mean age in years	% of Women	Intervention	Duration of intervention	Quality score
Berman et al.	Psychotherapy	2016	United States	21	37	49	100	“Accept yourself!” program based on Health At Every Size concept: - Integrates Acceptance and Commitment Therapy, mindfulness techniques - Psychoeducation - Focus on size acceptance and weight inclusivity - Workbook	11-week: 2 h per week	Total score: 15 - Reporting: 8 - External validity: 0 - Internal validity bias: 4 - Internal validity cofounding: 3 - Power: 0
Berman et al.	Behavior Therapy	2022	United States	19	Accept Yourself: 43.2 Control: 42.8	Accept Yourself: 52.7 Control: 50.3	100	“Accept yourself!” program based on Health At Every Size concept: (cf. above)	11-week: 2 h per week	Total score: 16 - Reporting: 7 - External validity: 0 - Internal validity bias: 5 - Internal validity cofounding: 4 - Power: 0
Carels et al.	*Annals of Behavioral Medicine*	2009	United States	46	36.6			LEARN manual (1 chapter per week): - Self-monitoring of eating behavior - Controlling stimuli associated with eating - Physical activity - Nutrition education - Modifying negative emotions associated with dieting - Setting realistic goals - Report diet and physical activity - Maintenance Manual OR Group Based Behavioral Weight Loss Program: - emphasized taking control of personal eating, - physical activity environment - enhancing motivation to weight loss	18-week: in two phases: Manual for 6 weeks then Group-based for 12 weeks, 90-min per week	Total score: 14 - Reporting: 7 - External validity: 0 - Internal validity bias: 5 - Internal validity cofounding: 2 - Power: 0
Carels et al.	Eating Behaviors	2010	United States	49	37.2	47.4	100	LEARN program: - self-monitoring of eating behavior - controlling stimuli associated with eating - physical activity - nutrition education - modifying negative emotions associated with dieting - setting realistic goals Transform Your Life program: - develop and maintain healthy habits and disrupt unhealthy habits - create a personal food and exercise environment that increases exposure to healthy eating and physical activity - facilitating participants’ weight loss motivation	14-week: 75-min per week	Total score: 11 - Reporting: 7 - External validity: 0 - Internal validity bias: 3 - Internal validity cofounding: 1 - Power: 0
Carels et al.	Journal of Health Psychology	2014	United States	59	39.7	44.3	78	New Perspective program: - disruption of unhealthy relationships to food - body image acceptance - becoming aware of and challenging internalized weight bias Transform Your Life program: - develop and maintain healthy habits and disrupt unhealthy habits - create a personal food and exercise environment that increases exposure to healthy eating and physical activity - facilitating participants’ weight loss motivation	12-week: 90 min per week	Total score: 17 - Reporting: 8 - External validity: 0 - Internal validity bias: 5 - Internal validity cofounding: 4 - Power: 0
Carels et al.	Eating Behaviors	2017	United States	38	35.7		87	Step One: - Self-help manual: one chapter per week - Record physical activity and energy intake Step Two: - Continue Self-Help book - Meal Replacement treatment (one pill per day) Step Three: Goal Miss - Continue meal replacement - Individual counselling intervention, 150 min per month: identification of barriers to weight loss success, learning problem solving skills, apply problem solving skills	24-week: 8 weeks per step	Total score: 11 - Reporting: 6 - External validity: 0 - Internal validity bias: 3 - Internal validity cofounding: 2 - Power: 0
Carels et al.	Journal of Contextual Behavioral Science	2019	United States	53	35.8	47.2	70	Step One: - Self-help manual: one chapter per week - Reach 500 steps per day Step Two: - Acceptance based behavioural treatment: 90 min meeting sessions per week.	16-week: 8 weeks per step	Total score: 12 - Reporting: 6 - External validity: 1 - Internal validity bias: 4 - Internal validity cofounding: 1 - Power: 0
Davies et al.	Body Image	2021	United States	135	Under 26	20.8	100	Expand your horizon: program centred on body functionality appreciation. Participants watched 3 videos over 5 days, and wrote during 10 min at each video for each day. Writing concerned gratitude about body functionality Control: same process but focused on college application	5 days	Total score: - Reporting: 8 - External validity: 0 - Internal validity bias: 5 - Internal validity cofounding: 4 - Power: 17
Forbes et al.	Journal of Cognitive Psychotherapy	2020	Australia	15	31.2	43.6	100	Compassion Focused Therapy: - 2 full day workshops one-week apart - 5 sessions per day focus on the development of self-compassion skills	2 days	Total score: 12 - Reporting: 6 - External validity: 1 - Internal validity bias: 4 - Internal validity cofounding: 1 - Power: 0
Haley et al.	Journal of Contextual Behavioral Science	2022	United States	15	37.3	34.7	100	Self-Compassion intervention: - Focuses on self-compassion applied to multiple areas of daily life - Home practice	3 weeks: two 1.5 h and a final 75 min session	Total score: 14 - Reporting: 7 - External validity: 1 - Internal validity bias: 3 - Internal validity cofounding: 3 - Power: 0
Levin et al.	Cognitive and Behavioral Practice	2018	United States	10	34.11	35.1	90	Guided Self-Help program: - Acceptance and Commitment Therapy for weight and weight stigma issues, - Addresses emotional and psychological factors as mindfulness, compassion, accepting unwanted emotion - Coaching to increase motivation, elicit change talk, reinforce successful adherence	7 weeks	Total score: 17 - Reporting: 7 - External validity: 0 - Internal validity bias: 5 - Internal validity cofounding: 5 - Power: 0
Levin et al.	Translational Behavioral Medicine	2021	United States	79	ACT: 34 Control: 33.5	ACT: 38 Control: 41		Combination of txt, video, interactive exercise Phone Coaching 5–10 min / week ACT skills: increase motivation, PA, Educational content”	8 weeks, 1 session/ week	Total score: - Reporting: 7 - External validity: 1 - Internal validity bias: 4 - Internal validity cofounding: 3 - Power: 0
Lillis et al.	Annals of Behavioral Medicine	2009	United States	84	ACT: 33.6 Control: 32.5	ACT: 49.8 Control: 51.7	90.5	Acceptance and Commitment Therapy workshop focus on: - Weight stigmatization - Acceptance and mindfulness - Defusion skills applied to difficult situation - Workbook	1 day, 6 h	Total score: - Reporting: 9 - External validity: 1 - Internal validity bias: 5 - Internal validity cofounding: 4 - Power: 1
Mensinger et al.	Appetite	2016	United States	80	WNP: 37.4 CWMP: 38.5	WNP: 39.8 CWMP: 39.3		Weight Neutral Program: - Body size acceptance - Self-Affirmation development - Social support - Booklet: psychoeducation - Apply what has been learn at home Conventional weight management program: LEARN Program (cf. above)	6 months: 90 min per week	Total score: 18 - Reporting: 8 - External validity: 0 - Internal validity bias: 5 - Internal validity cofounding: 4 - Power: 1
Myre et al.	Applied Psychology: Health and Well-Being	2020	Canada	103	IR: 45 Control: 41	IR: 45 Control: 44		Implicit Retraining Task: the task consists in pairing counter-stereotypical images of active individuals with obesity with positive physical activity-related words (e.g., energetic, healthy etc.). New Images were introduced each week to avoid over-familiarisation Control: read the read Canada’s Physical Activity Guidelines for adults	3 weeks, 1 session/week	Total score: 19 - Reporting: 9 - External validity: 0 - Internal validity bias: 5 - Internal validity cofounding: 4 - Power: 1
Olson et al.	International Journal of Eating Disorders	2018	United States	32	Body Project: 29.5 Control: 30.6	Body Project: 41.9 Control: 40.6	100	Standard: - Psychoeducation - Recommendation about nutrition and physical activity Body project: body acceptance program. - Improve body image by critiquing unrealistic ideals beauty. - Challenging the system and confront stigma - Prioritize Self care	4-week: 1 h per week	Total score: 18 - Reporting: 6 - External validity: 0 - Internal validity bias: 7 - Internal validity cofounding: 4 - Power: 1
Palmeira et al.	Journal of Health Psychology	2019	Portugal	53	34.1	42.5	100	KG-Free intervention: - Psycho education (eating and emotion) - Mindfulness skills - values clarification - Acceptance and defusion skills - Self-compassion skills	10-week: 2,5 h per week	Total score: 19 - Reporting: 9 - External validity: 0 - Internal validity bias: 6 - Internal validity cofounding: 3 - Power: 1
Palmeira et al.	Appetite	2017	Portugal	73	KG-free: 34.8 TAU: 33.6	KG free: 41.9 TAU: 42.7	100	KG-Free intervention: (cf.above) Treatment as usual: - Medical and nutritional appointment - Physical examination - Tailored diatery recommendation - PA prescriptions - No psychological intervention	10-week: 2,5 h per week	Total score: 21 - Reporting: - External validity: 1 - Internal validity bias: 6 - Internal validity cofounding: 4 - Power: 1
Pearl et al.	Eating and Weight Disorders	2016	United States	18	Intervention: 42.2 Control: 38.6	Intervention: 54 Control: 52.7		Weight BIAS Program: - weight-related cognitive distortions - thought records - cognitive restructuring and reappraisal - assertiveness training - empowerment - body acceptance improvement	8-week: 1 h per week	Total score: 16 - Reporting: 8 - External validity: 0 - Internal validity bias: 4 - Internal validity cofounding: 4 - Power: 0
Pearl et al.	Journal of Consulting and Clinical Psychology	2020	United States	72	BWL: 38.4 BWL + BIAS: 40.1	BWL: 46.6 BWL + BIAS: 47.7	84.7	Behavioral Weight Loss program (BWLP): - eating behaviour, body image, and emotional management - Self-Monitoring - social support - goal setting - physical activity prescription + - recipe discussion Weight BIAS program: same as BWLP content combined with a weight stigma intervention: - Acceptance and Commitment Therapy - Psychoeducation about Weight and weight bias - Challenging myths and cognitive distortion - Restructuring Neg thoughts and - reappraising Stigmatized situations - Self-acceptance and Mindfulness skills development	12-week: 90 min per week	Total score: 21 - Reporting: 11 - External validity: 0 - Internal validity bias: 5 - Internal validity cofounding: 4 - Power: 1
Pearl et al.	Obesity	2020	United States	72	BWL: 38.4 BWL + BIAS: 40.1	BWL: 46.6 BWL + BIAS: 47.7	84.7	BWLP: (cf. above) Weight BIAS program (same as BWL content but combined with a weight stigma intervention): - Acceptance and Commitment Therapy - psychoeducation about Weight and weight bias - Challenging myths and cognitive distortion - restructuring Neg thoughts and - reappraising Stigmatized situations - self-acceptance and Mindfulness skills development	90 min groups meeting 12 weekly group sessions 6 months after the end of the program	Total score: 20 - Reporting: 10 - External validity: 0 - Internal validity bias: 5 - Internal validity cofounding: 4 - Power: 1
Potts et al.	Behavior Modification	2022	United States	55	GSH-P: 36.7 GSH_E: 36.6 WL: 37.8	GSH-P: 37.4 GSH_E: 35.9 WL: 42.8	82.1	Guided Self Help (GSH) book: - One chapter per week - Acceptance and Commitment Therapy to reduce harmful effect of weight stigma - develop more adaptative behaviors GSH-E: - receive mail once a week to remind the task of the week - brief tailored supportive statement. GSH-P: - receive mail + weekly phone call - promote engagement with self-help materials. - enhancing motivation - Helping to generalize skills to daily life	7 to 8 weeks	Total score: 16 - Reporting: 8 - External validity: 0 - Internal validity bias: 5 - Internal validity cofounding: 3 - Power: 0
Scagliusi et al.	Human Organization	2020	Brazil	39	I-HAES: 34.9 Control: 34.5	I-HAES: 32.9 Control: 37	100	Intensive Health At Every Size program (I-HAES): - physical activity program - nutritional sessions: discussion about hunger, satiety, appetite, pleasure and emotional eating - food diary - 5 philosophical workshops CTRL: followed the traditional HAES program with a bimonthly educational lecture about adoption of healthy lifestyle	7-months	Total score: - Reporting: 7 - External validity: 0 - Internal validity bias: 3 - Internal validity cofounding: 3 - Power: 0
Wallin et al.	Journal of Contextual Behavioral Science	2018	Sweden	13	29.9	42	100	Self-help intervention using a workbook based on acceptance and commitment therapy (ACT). Three chapters following key processes in ACT were proposed: values, acceptance and mindfulness, Committed Action	3-week: 1 h per week	Total score: - Reporting: 7 - External validity: 0 - Internal validity bias: 6 - Internal validity cofounding: 4 - Power: 0

### Methodologies

Of the 24 studies, 23 were interventional, involving a behavioral weight-loss (BWL) program, cognitive-behavioral therapy (CBT), or a combination of these two types of programs, with pretreatment and posttreatment analyses and follow-up. All these studies were longitudinal and used quantitative measures. Only one study, based on a content analysis of interviews with patients who had taken part in an interventional study, was qualitative. Ten of the 24 studies used an intervention that did not directly target the adverse effects of weight stigma but explored the influence of the intervention on weight stigma outcomes. Eight of the 24 studies proposed an intervention that directly targeted the adverse effects of weight stigma and investigated its effect on quality of life. Finally, the remaining six studies targeted potential mediators of the deleterious effect of weight stigma on quality of life.

### Rigor and quality of the studies

A summary of the quality scores is displayed in Table 1. The mean quality score was 16.5. The quality assessment of the 24 studies highlights that four of them were good quality, fourteen were fair quality and six were low quality. The scores were very low in external validity, internal validity—confounding and statistical power. This indicates that the studies had the insufficient statistical power to detect important effects and none of the findings could be generalized to older population. Serious risk bias can be highlighted such as small samples, majority of one gender, no comparison or control group.

### Outcomes

The results of the studies included in the review are set out in Table 2. Most studies (92%) observed the effect of a group intervention or self-help book or both on quality of life, while 54% looked at the maintenance of beneficial effects after the end of the intervention. The most frequently assessed psychosocial factors were weight bias internalization (WBI) (96%), depressive symptoms (50%), and body image concerns (41%). In this study, we looked at the effects of interventions on these outcomes.

**Table 2:** Results of studies included in review.

Author	Objective and method assessment times	Data collection	Summary statistics	Results
Berman et al. (2016)	Provide initial evidence about the feasibility and safety of a brief, manualized ACT and HAES intervention for women living with obesity with depression Quantitative: questionnaires Pre- and Post-treatment and 3 months follow-up	- Depressive symptoms assessed with the Patient Health questionnaire and the Hamilton Rating Scale - Quality of life assessed with the Obesity-related Well-being questionnaires - Weight Stigma assessed with Weight Self Stigma Questionnaire - Body Image assessed with the body image acceptance and action questionnaires - Physical activity (PA) assessed with The Stanford Seven-Day Physical Activity Recall - Vital Signs: Blood pressure was assessed	- Repeated Measures Analysis of variance (ANOVA) - Simulation Modelling Analyses (SMA)	- Significant improvement during treatment and sustained after a brief follow-up: ○ Less depressive symptoms ○ Higher Obesity related quality of life ○ Lower level of Weight Self-stigma ○ Higher body image and acceptance ○ Higher frequency of PA practice - Improvement occurred without weight loss - Program feasible and safe - Low level of attrition (14%)
Berman et al. (2022)	Conduct a Randomized control Trial comparing Accept-Yourself (AY) to a group-based behavioural weight loss program Weight Watchers (WW). Investigate effects of the intervention over a 1-year follow-up Quantitative: Questionnaires + physical test and + metabolic measures Pre- and Post-treatment and follow-up (3, 6, 9 and 12 months)	- Depression assessed with Patient Health Questionnaire and Hamilton Rating Scale for Depression - Cardiovascular fitness assessed with the 6-min walk test - Quality of life assessed with the Obesity-related Well-being questionnaires - Weight Stigma assessed with Weight Self Stigma Questionnaires - Body Image assessed with the body image acceptance and action questionnaires - Eating behaviour assessed with The Eating Disorder Diagnostic Scale - Metabolic measures: Blood pressure, Metabolic Health	- Linear Mixed-effects model for repeated measures - Analyses of variances within and between participant - Independent samples t-test - Chi-square	- Both groups improved their level of depression (decreased symptoms) - AY improved Obesity-related quality of life, Eating Behaviors and Body Image - Weight Self Stigma: significant interaction Group × Time. The AY group decrease their level of Weight self-stigma during intervention but returned to a level equivalent to pre-treatment after one year’s follow-up - WW program worsened all outcomes in general, particularly Eating Behaviors and Weight
Carels et al. (2010)	Examine existence of the relationships among explicit, implicit and internalized weight stigma in a sample of individuals with overweight or obesity seeking weight loss treatment. Quantitative: Questionnaires + Physical measure Pre- and Post-treatment (14-week)	Weight stigma assessed with: - Obese Persons Trait Survey (explicit bias) - Implicit associations test (Implicit Bias) - Weight Bias Internalization scale Body Image assessed with tow subscale of the Multidimensional Body Self Relations Questionnaire: - Appearance Evaluation (AE) - Appearance Orientation (AO) Binge Eating assessed with the Binge Eating Scale Depression assessed with the Center for Epidemiological Studies-Depression scale	- Paired-sample t-test - Pearson correlations	- Improvement in all outcomes from pre- to post-treatment - Greater baseline weight associated with greater internalized weight bias - Implicit weight bias unrelated to explicit and internalized weight bias - Greater change in internalized weight bias from pre-to post-treatment was correlated to greater change in AE - Significant decreases in internalized weight bias, as well as both positive and negative attributions of people living with obesity, were observed in the current investigation.
Carels et al. (2014)	Compare treatment outcomes (e.g., weight loss, self-monitoring, psychological variables) between the New Perspective (NP) (a novel program) and the Transform Your Life program (TYL) (validated program). Quantitative: Questionnaires + BMI + Caloric Intake Measures at Pre-, Post-treatment and 6 months follow-up	Eating behaviors assessed with the Binge Eating Scale, The Emotional Eating scale and the Housefold food inventory Body Image assessed with the Multidimensional Body Self Relations Questionnaire Weight Stigma assessed with the Weight Bias Internalization Scale and the Obese Persons Trait Survey Self-objectification assessed with the Trait Self-objectification questionnaire	- 2×2 repeated measures analyses of variance - Multivariate Analysis of Variance - Within subjects ANOVAs	From pre-to post-treatment Both groups: - Lost significant amount of weight - Improved Body Image, Emotional and Binge eating - Significant decrease in Weight bias internalization - Attribute lower negative traits to themselves From post-treatment to 6 months follow-up: TYL group continued to lose significant amount of weight while NP group regain weight
Carels et al. (2019)	Examine the effectiveness of a Stepped Care Acceptance Based Therapy (ABT) Approach, during 16-week, to weight loss for individuals who did not meet their weight loss goal with a Self-Help behavioral weight loss treatment and to examine predictors of poor treatment outcome Quantitative: Questionnaires + BMI Pre-treatment, 8 week and 16 week (Post-treatment)	- Eating behaviours assessed with the Binge eating scale, the Food acceptance and Action Questionnaire and the Cognitive Fusion Questionnaire - Weight stigma assessed with the Weight bias internalization scale - Depression assessed with the Center for Epidemiological Studies-Depression scale - Life values assessed with the Valuing Questionnaire - Health literacy assessed with the Newest Vital Sign	- A mixed within and between repeated measures ANOVA - Paired samples t-tests - Pearson correlation	- Weight Bias Internalization Score: ○ No interaction for time and condition ○ Decreased score across time ○ Lower level of Weight bias internalization at pre-treatment and Post-treatment for participants who achieve their weight loss goal - Participants who achieved their weight loss goal in the first stage were those who benefited most from the programme and improve in all outcomes - Participants who did not reach their weight-loss goal in the first step had higher levels of weight bias internalization and depression before treatment
Carels et al. (2017)	Examine the effectiveness of a stepped-care behavioral weight loss treatment Examine factors that contribute to poor weight loss outcomes and the for more intensive treatment Quantitative: Questionnaires + BMI Pre-treatment, at 2 and 4 months of treatment and post-treatment (6^th^ month)	- A special device for measuring the number of steps taken each day was used to assess energy expenditure - An online dashboard was used to assess self-monitoring of weight and diet - Health literacy assessed with the Newest Vital Sign - Weight stigma assessed with the Weight bias internalization scale and the Universal Measure of Bias-FAT - Binge eating assessed with the Binge eating scale	- Paired sample t-tests - Repeated measures analysis of variance (RANOVA) - One-way analysis of variance (ANOVA) - Independent samples t-tests	- Significant interaction between steps status and weight loss trajectory: Unstepped participants lost more weight than the others - No effect of weight bias internalization on stepped status, self-monitoring and the other psychological factors - Only health literacy was related to the success of the interventions: unstepped participants, who had higher level of health literacy, had low level of attrition, managed their calories better and had no difficulties to understand concepts in weight loss manual than Once and twice stepped participants
Carels et al. (2009)	Examine the relationship between weight bias and treatment outcomes, such as caloric intake, exercise and weight loss. Quantitative: Questionnaires, Caloric intake and Weight	Weight stigma assessed with: - Obese Persons Trait Survey (explicit bias) - Implicit associations test (Implicit Bias) Caloric intake assessed with a daily diaries use to record all food intake Energy expenditure assessed with an accelerometer device	- Analysis of variance (ANOVA) - Chi-square analyses - Pearson correlation	- During Self-help phase: ○ Lower energy expenditure and greater caloric intake was related to attributing more negative traits and less positive traits to people with obesity. ○ When participants attributed more positive traits to individuals with obesity the percentage of weight loss was higher - At the end of the program: ○ participants with greater frequency of self-monitoring attributed more positive traits to persons with obesity ○ Weight bias was not associated with weight loss
Davies et al. (2021)	Examine the effectiveness of a body gratitude journaling intervention (i.e., Expand your Horizon: EYH) compare with an active writing condition (i.e., expressive writing) Quantitative: Questionnaires Pre- and post-treatment and one-week follow-up	- Weight bias assessed with the Modified Weight bias internalization scale - Self-compassion assessed with the Self-compassion scale-short form - Body appreciation assessed with the functionality appreciation scale - Health related attitudes assessed with the Healthcare Stress Scale	- Linear Mixed Models (LMMs)	- Overall, all outcomes improved in both condition but the magnitude of changes was greater for participants who followed the Expand Your Horizon intervention at post-treatment and follow-up - Significant time × condition interaction: Weight bias internalization decreased more at both time points in the EYH condition. - The EYH condition showed significantly greater decrease in Healthcare stress over time at one-week follow-up
Forbes et al. (2020)	Develop an intensive, 2-day, compassion focused therapy-based group program targeting weight stigma in women with overweight or obesity, and determine the feasibility and acceptably of this approach. Quantitative: Questionnaires Pre-treatment, Post-treatment (i.e., 2 days) and follow-up (i.e., 3 months)	- Self-compassion assessed with the Self-Compassion Scale - Weight Stigma assessed with the Weight Bias Internalization Scale and the Stigmatizing situations Inventory-Brief - Psychological distress assessed with the Depression and Anxiety Stress Scale-short form - Quality of life assessed with the Satisfaction With life Scale and the UCLA Loneliness Scale. - Body Image assessed with the Weight and Body-Related Shame and Guilt Scale and a modified version of the Body Image Concern Subscale - Self-efficacy assessed with the Weight Efficacy Lifestyle Questionnaire - Perception of the intervention assessed with the Credibility Scale	- One-way repeated measures analysis of variances - Pairwise comparison	- Significant improve in self-compassion and internalized weight stigma from pre- to post-treatment but not from post-treatment to 3 months follow-up (Large effect size) - Significant improvement, from pre to post-treatment with large effect size for psychological distress, quality of life, body image and self-efficacy - Only body dissatisfaction continued to significantly improve during the follow-up period
Haley et al. (2022)	Examine aspects of feasibility and acceptability of a 3-sessions self-compassion intervention for women with overweight/obesity and internalized weight stigma. Quantitative: Questionnaires Pre-treatment and Post-treatment (i.e., 3 weeks)	Self-Compassion assessed with the Self-Compassion Scale Weight Stigma assessed with the Weight Bias Internalization scale Body Image assessed with the Body Image Shame Scale and the Body Appreciation Scale Eating Behaviors assessed with two subscale of the Three Factor eating Questionnaire (i.e., Emotional Eating and Uncontrolled Eating) and the Intuitive Eating Scale.	- Paired samples t-tests	From pre to post-intervention: - No significant effect for the level of weight stigma et the body image shame - Large effect size for intuitive eating and body appreciation - Medium effect size for Uncontrolled Eating and Emotional Eating - Small effect size for Self-compassion - High engagement and low attrition rate observed due to the duration of the intervention
Levin et al. (2021)	Evaluate an Acceptance and Commitment Therapy (ACT) on Health, which integrate nutrition education, physical activity goal setting and an 8-session online guided self-help course with weekly phone coaching. Quantitative: Questionnaires + Physical measures Pre-treatment, Post-treatment (i.e., 8 weeks) and Follow-up (i.e., 8 weeks)	- Eating Behaviors assessed with the Automated Self-administered 24-h Recall, the Three Factor Eating Questionnaire (TFEQ) and the Weight Control Strategy Scale - Weight Stigma assessed with the Weight Self-Stigma Questionnaire - Physical Activity assessed with the International Physical Activity Questionnaire - Participants self-reported their weight - Mental Health assessed with the General Health Questionnaire - Psychological inflexibility assessed with Acceptance and Action Questionnaire for Weight	- Analysis of Covariance - Paired sample t-tests - Mediational analyses - Bootstrapping	- Significant condition effects: Participants assigned to ACT program improve more on healthy eating, self-reported weight, mental health, weight stigma and psychological inflexibility, but not on self-reported physical activity - Better mental health was related to completing more sessions - From post-treatment to 8-week follow-up: the Automated Self-administered 24-h Recall worsened and cognitive restraint with eating (TFEQ) improved. - Psychological inflexibility mediated the effect of ACT program on the Three Factor Eating Questionnaire and the Weight Self-stigma Questionnaire - Participants found phone coaching very useful because it maintained a high engagement rate
Levin et al. (2018)	Evaluate the preliminary feasibility and efficacy of a guided self-help Acceptance and Commitment Therapy (ACT) Intervention for individuals with overweight or obesity high in weight self-stigma Quantitative: Questionnaires + Physical measures	- Weight Stigma assessed with the Weight Self-Stigma Questionnaire - Eating Behaviors assessed with the Dutch Eating Behavior Questionnaire - Weight Management assessed with the Weight Control Strategies Scale, the Motivating Factors for Weight Loss and a digital scale - Quality of life assessed with the Global Health Scale - Depression assessed with Patient Health Questionnaire - Valued actions assessed with the Valuing Questionnaire - Psychological Inflexibility assessed with the Acceptance and Action Questionnaire for Weight	- Mixed Model Repeated Measures - Chi-square analyses - Independent Sample t-tests	- Significant improvement from pre to post-treatment, with large effect size, for weight self-stigma, weight management, depression, eating behaviors, quality of life, physical activity - No significant differences between post-treatment and 3-month follow-up for almost all outcomes, only the subscale Fear of enacted stigma of the Weight Self-Stigma Questionnaire continued to improve. - Significant improvement over time, with large effect size, for processes of change (i.e., psychological inflexibility and valued action) - Significantly greater improvements in psychological inflexibility, values progress and mental health among participant assigned to the coach allowing discussions of issues with greater depth
Lillis et al. (2009)	Examine whether a 1-day Acceptance and Commitment Therapy workshop, focus on weight stigma, could improve general mental health, quality of life and weight self-stigma, while also augmenting weight control efforts by increasing acceptance, mindfulness and values-based action Quantitative: Questionnaires + Physical measures	- Quality of life assessed with the Obesity-related Well-being questionnaires - Weight Stigma assessed with the Weight Self-Stigma Questionnaire - BMI was calculated from objectively measured height and weight. - Psychological Inflexibility assessed with the Acceptance and Action Questionnaire for Weight - Distress Tolerance assessed with a breath-holding test	- Analysis of Covariance - Mediational analysis - Paired sample t-tests	- Significant improvement in all outcomes from pre to post treatment - Improvement sustained at 3 month follow-up - Post hoc analysis suggest that the improvement of stigma, distress and quality of life was due to the effect of the ACT intervention and not due to the weight loss - Participant who read the workbook more often showed greater improvement on all outcome
Mensinger et al. (2016)	Examine the moderating effect of internalized weight stigma on eating behavior outcomes over time when comparing a weight neutral program to a conventional weight-management program for women with high BMI Quantitative: Questionnaires Pre-treatment, Post-treatment (i.e., 6 months) and follow-up (24 months)	- Eating behaviors assessed with Intuitive Eating Scale and the Eating Disorder Examination Questionnaire - Weight Stigma assessed with Weight Bias Internalization Scale	- Independent samples t-tests - Chi-square tests - Linear Mixed Models	- Both groups significantly decreased their level of Weight Bias Internalization (WBI) at post-treatment with no difference between them - Both groups sustained positive changes in WBI at 24 months follow-up. - Participants with high level of weight bias internalization got poorer improvement in Disorder eating and intuitive eating, while lower level of weight bias internalization was related to greater improvement
Myre et al. (2020)	Reduce Weight Bias Internalization by utilizing an implicit retraining task that paired counter-stereotypical images of active individuals with obesity and positive physical activity words Quantitative: Questionnaires Pre-treatment, Post-treatment (i.e., 3 weeks) and Follow-up (i.e., 1 week)	- Weight stigma assessed with the Weight Bias Internalization Scale and the brief Stigmatizing Situations Inventory - Physical Activity assessed with a Go/No Go Association Task, a 7-point bipolar adjective scale, PA self-efficacy scale and the Godin-Shepherd Leisure Time Exercise Questionnaire	- Repeated-measures analysis of variance - Pearson’s Correlations	- Effect of Group on WBI: Lower WBI at post treatment for the implicit retraining group - No time by group interaction on WBI - Difference in WBI over time when participants of both groups were combined - No significant difference nor interaction of the implicit retraining task on PA outcomes from pre-to post-treatment
Olson et al. (2018)	Replicate the Body Project Treatment effect when combine with a behavioral weight loss recommendations in a novel sample of women with overweight or obesity Quantitative: Questionnaires + Physical measures Pre-treatment, Post-treatment (4 weeks)	- BMI calculated by measuring height and weight - Body Image assessed with the Sociocultural Attitudes Toward Appearance Questionnaire, the Body Shape Questionnaire and the Body Appreciation Scale	- Repeated-measures Analysis of Variance	- Both conditions improved Body Dissatisfaction, Weight Bias Internalization and BMI - Group × Time interaction: Greater improvement for the group that combined the Body Project with a behavioral weight loss recommendation
Palmeira et al. (2019)	Examine the effectiveness of the KG-Free intervention at post-treatment and 3 month follow up and explore the psychological processes that underlie changes observed on outcomes Quantitative: Questionnaires + physical measures Pre-treatment, Post-treatment (12 week), 3-month follow-up	Main outcome measures: - Height and Weight - Weight Self-Stigma Questionnaire - Obesity Related Well Being Questionnaire - Thee Factor Eating Questionnaire Mediator processes: - Acceptance and Action Questionnaire - Others as Shamer Scale - Self-Compassion Scale - Five Facet Mindfulness Questionnaire	- Repeated measures analysis of variances - Mediation and moderation analysis for repeated measures designs - Serial Mediation Model	- At Post-treatment: ○ Increased Quality of life, mindfulness and self-compassion abilities ○ Decreased BMI, weight self-stigma, weight related experiential avoidance, shame and self-judgment levels ○ These changes were maintained at 3-month follow-up - Direct effect of intervention on Quality of life was non-significant - Quality of life increased through indirect effect: ○ Development of mindfulness abilities ○ Decrease in Weight self-stigma; Weight related experiential avoidance, shame and self-judgment
Palmeira et al. (2017)	Test the efficacy of a group intervention (i.e., KG-Free) for women with overweight or obesity based on mindfulness, ACT and compassion approaches Quantitative: Questionnaires + physical measures Pre-treatment, Post-treatment (12 week)	Main outcomes measures: - Weight Self-Stigma Questionnaire - Obesity Related Well Being Questionnaire - Thee Factor Eating Questionnaire Secondary outcomes measures: - Height and Weight - Waist circumference - Blood samples - General Health Questionnaire Process measures: - Acceptance and Action Questionnaire - Forms of Self-Criticizing & Self-Reassuring Scale - Self-Compassion Scale - Five Facet Mindfulness Questionnaire	- Independent sample t test - Analysis of Covariances - Paired samples t-tests	- At baseline, the intervention group had higher levels of weight-related experiential avoidance, self-criticism and a diminished quality of life - Intention to treat: ○ Increased quality of life and decreased in weight self-stigma, emotional and uncontrolled eating in both groups ○ Physical Activity and Self-compassion abilities increased in the intervention group ○ Psychological distress and hated self decreased in the intervention group - Intervention efficacy: ○ Between groups: Intervention group improved more in weight-self stigma, emotional and uncontrolled eating, quality of life and practiced more physical activity in a week ○ Within groups: significant difference from baseline to post-treatment in all outcomes only for the intervention group
Pearl et al. (2018)	Tested a novel group-based, cognitive-behavioral intervention design to reduce internalized weight stigma among individuals with obesity Quantitative: Questionnaires Pre-treatment, post-treatment (8 week)	Weight Stigma assessed with the Weight Bias Internalization Scale and the Fat Phobia Scale Self-efficacy assessed with the Weight Efficacy Life Style Questionnaire Depression symptoms assessed with the Beck Depression Inventory	- Multivariate analysis of variance	- Average weight remained stable - Significant decrease in Weight Bias Internalization and Fat phobia scale for the intervention group compare to control group - Significant increase in Self-efficacy in the intervention group compare to the control group - No between groups difference in depression symptoms
Pearl et al. (2020a)	Investigate the effects of a cognitive-behavioral intervention for weight bias internalization combined with behavioral weight loss Quantitative: Questionnaires Pre-treatment, post-treatment (12 week) and 3-month follow-up	Primary outcome: - Weight Bias Internalization Scale - Weight Self-Stigma Scale - The Fat Phobia Scale Secondary Outcomes: - Impact of Weight on Quality of Life Questionnaire-Lite - Patient Health Questionnaire - Body Appreciation Scale - Weight and Lifestyle Efficacy Short Form - Self-Efficacy for Exercise Scale - Eating Inventory	- Chi-square analysis - Analysis of Variance - Linear Mixed Models	- Significant reduction in all weight stigma measures across group - No difference between groups in Weight Bias Internalization at post-treatment - Significant reduction in Weight Self-Stigma scale for the intervention group. This effect is due to the difference in the Self-devaluation subscale - Changes maintained à 3-month follow-up - Improvement in all secondary outcomes for both groups
Pearl et al. (2020b)	Investigate the effect of a 6-month non-intervention follow-up effect of a cognitive behavioral intervention for weight bias internalization combined with behavioral weight loss Quantitative: Questionnaires Pre-treatment, 3-month follow-up and 6-month follow-up	Primary outcome: - Weight Bias Internalization Scale - Weight Self-Stigma Scale - The Fat Phobia Scale - Coping with Weight Stigma Scale Secondary Outcomes: - Impact of Weight on Quality of Life Questionnaire-Lite - Patient Health Questionnaire - Body Appreciation Scale - Weight and Lifestyle Efficacy Short Form - Self-Efficacy for Exercise Scale - Eating Inventory	- Linear Mixed Models - Analysis of Variance	From Pre-treatment to 6-month Follow up: - Significant reduction in all weight stigma measures across group - No between group difference in Weight Bias Internalization and Weight Self-Stigma Scale - Significant reduction in negative affect following experiences of weight stigma in the intervention group - Improvement in depression, body image and Quality of life across group - Greater acquisition and use of skills for the intervention group
Potts et al. (2022)	Evaluate an Acceptance and Commitment Therapy guided self-help book with guidance from phone coaching or brief email prompts Quantitative: Questionnaires + Physical Measure Pre-treatment and post-treatment (8-week)	Main outcomes measures: - Weight Self-Stigma Questionnaire - Weight Control Strategies Scale - Dutch Eating Behavior Questionnaire-Emotional Eating - Eating Disorder Examination Questionnaire-Binge Eating Episodes - International Physical Activity Questionnaire-Short Form - Height and Weight Process of changes measures: - Acceptance and Action Questionnaire for Weight-Related Difficulties - Comprehensive Assessment of Acceptance and Commitment Therapy Processes - Series of self reported items relative to self reported use and satisfaction with the self-help book	- One Way analysis of variance - Chi-square - T-tests - Mixed Model Repeated Measures analyses	- No difference in reading the guided self help book between groups - Significant time by condition interaction in groups who received phone coaching and email prompts compared to the control group: ○ Lower level of Weight Self-Stigma ○ Better coping strategies relative to weight control, physical activity and diatery choice ○ Lower psychological inflexibility
Scagliusi et al. (2020)	Qualitatively describe the responses to weight stigma and body acceptance issues from urban Brazilian Gorda Women who participated in two forms of Health at Every Size intervention Qualitative: Verbatims	Authors used an interview grid focused on Body Acceptance issues and Response to Weight Stigma		- About Body Acceptance Issues, the group who received the intensive version of the intervention evaluate themselves better and change in the perception of oneself as a person. They promote acceptance of oneself and engaged in new experiences while the control group felt resign and unaccepted their body - About Response to weight stigma, the group who received the intensive version of the intervention do not care about the judgment of other. They learned how to challenge and deconstruct the norm and expectations expressed by others. The control group took stereotypes lightly or made jokes to deal with it.
Wallin et al. (2018)	Evaluate the impact of a brief self-help Acceptance and Commitment Therapy (ACT) intervention to improve value attainment related to health and decrease weight related experiential avoidance Evaluate the acceptability of the intervention Quantitative: Questionnaires Daily, Pre treatment, Post-treatment (3-week) and 3-month follow-up	Outcomes measure collected daily: - Bull’s Eye Value Survey - Willingness to Pay-Distress Intolerance Outcomes measures at pre-treatment, post-treatment (3-week) and 3-month follow-u: - Acceptance and Action Questionnaire for Weight-Related Difficulties - Brunnsviken Brief Quality of Life Inventory - Weight Self-Stigma Questionnaire - Hospital Anxiety and Depression Scale - Treatment Acceptability and Anticipated Adherence Scale	- Two tailed Wilcoxon signed-rank tests	- Development of Acceptance, Commitment and Mindfulness skills through the usage of the self-help book - Significant increased general quality of life at post treatment - Significant Decreased level of experiential avoidance, weight self-stigma, symptoms of anxiety and depression with a large effect size

### Presentation of results

All the results of this literature review are presented in the same way: a brief description of the studies’ objectives, followed by a description of the specific features of the proposed programs and a description of the effects observed on weight stigma. We have reported all the results: those indicating that remediation of the deleterious effects of stigmatization contributes to a better quality of life and those refuting this hypothesis.

### Interventions directly focused on weight stigma

Fourteen of the 24 studies proposed an intervention that directly targeted obesity stigma or self-stigma (Carels et al., 2014; Davies et al., 2021; Forbes et al., 2020; Levin et al., 2018; Lillis et al., 2009; Myre et al., 2020; Olson et al., 2018; Palmeira et al., 2017, 2019; Pearl et al., 2018, 2020a,b; Potts et al., 2022; Scagliusi et al., 2020). Among these studies the duration was different and short-term interventions and long-term interventions were identified.

Firstly, interventions lasting less than 4 weeks were designated as short-term and were analyzed. One study proposed a one-day intervention (Lillis et al., 2009), resulting in a marked decrease in stereotype levels and psychological distress and an increased level of quality of life. Moreover, *post hoc* analyses indicated that the benefits were not due to weight loss but rather to the content of the intervention. Forbes et al. (2020) underlined the maintenance of the beneficial effects of an intensive two-day program, notably through a reduction in the level of stereotype internalization and an increase in the level of self-compassion. These improvements were linked to improvements in other determinants, such as body image satisfaction and self-efficacy related to eating. Participants lost weight, and the authors speculate that this was due to the implementation of healthier behaviors; however, these behaviors were not evaluated. Finally, Myre et al. (2020), who proposed an online implicit retraining task, highlighted the difficulty of deconstructing automatic associations linked to obesity (i.e., associating obesity with a lack of physical activity). After 4 weeks of implicit retraining, the internalization level of the experimental group did not decrease. Moreover, the difference in WBI levels between the control and experimental groups is explained by an initial difference and not by an effect of the intervention.

Six Interventions lasted more than 4 weeks and were then classified as long-term and analyzed. Two of these six interventions offered participants a guided self-help (GSH) book that is based on acceptance and commitment therapy (i.e., *The Diet Trap*) combined with telephone coaching (Levin et al., 2018; Potts et al., 2022). The GSH book helped to reduce the level of WBI. Behaviorally, participants developed better weight-control strategies. It seems that telephone coaching contributed to a better assimilation of the skills acquired with the GSH book (Levin et al., 2018). Coaching not only kept participants motivated but also provided them with regular feedback on the GSH book (Potts et al., 2022). As a result, they better understood how to apply the skills they had acquired to everyday situations.

The other four studies evaluated the benefits of a program targeting WBI. The New Perspective (NP) program (Carels et al., 2014) teaches participants to become aware of and challenge internalized weight biases. This method was shown to reduce participants’ negative self-evaluation, encouraging greater acceptance of their body image. In addition, participants felt more able to manage their emotions when faced with difficult situations and developed healthy habits in their relationships to food and physical activity. Nevertheless, 6 months after the end of the NP program, the participants regained almost as much weight as they had lost, suggesting that the acquired skills are less useful for maintaining a stable weight. In the other three studies, the benefits of the Weight Bias Internalization and Stigma (Weight BIAS) program (Pearl et al., 2018, 2020a,b) were analyzed. In a first pilot study, Pearl et al. (2018) observed a decrease in the level of WBI and an increase in self-efficacy related to eating. Participants’ weight remained stable during the intervention, suggesting that the benefits were indeed due to the program and not to weight loss. However, a lack of follow-up after the intervention meant that it was not possible to determine whether the beneficial effects persisted over time. Pearl et al. (2020a), therefore, proposed a longer intervention (i.e., 12 weeks versus 8 weeks in the pilot study). One group followed a conventional BWL program, while another group followed the same program in combination with Weight BIAS (BWL + BIAS). Both programs reduced the level of WBI, but the reduction was greater with the combined program. More precisely, when we analyzed the subscales of the tool used (i.e., Weight Self-Stigma Questionnaire), the combined group had a lower score in terms of self-devaluation, which is the tendency to denigrate oneself. Intragroup analyses also revealed significant changes, including greater self-efficacy in weight and lifestyle management, fewer depressive symptoms, and greater body appreciation. These beneficial effects were maintained 6 months after the end of the program (Pearl et al., 2020b). No significant intergroup differences were reported, meaning that overall, both groups benefited equally from their respective programs. Nevertheless, the mechanisms of action appear to differ between the two groups. According to the qualitative analysis (Pearl et al., 2020b), participants in the BWL + BIAS group reported more self-appreciation and self-esteem and implemented more coping strategies in the face of stigmatizing contexts, whereas participants in the BWL group reported feeling less lonely and more socially supported. These factors may all contribute to improved quality of life and reduced WBI. In addition, the authors explained that the lack of difference between the groups was due to an insufficient dose of the intervention targeting obesity stigma. Thus, it would be relevant to propose a longer and denser intervention addressing these issues to detect any specific effects of an intervention of this kind.

### Interventions indirectly addressing weight stigma

Six of the 24 studies (Carels et al., 2014; Davies et al., 2021; Olson et al., 2018; Palmeira et al., 2017, 2019; Scagliusi et al., 2020) focused on determinants mediating the adverse consequences of obesity stigma. Five of the six studies were quantitative, and one was qualitative (Scagliusi et al., 2020).

Body preoccupation has emerged as a relevant determinant. Three of the six studies focused on the body, more specifically on body image (Olson et al., 2018), body functionality (Davies et al., 2021), and body appreciation (Scagliusi et al., 2020). The Body Project program (Olson et al., 2018) criticizes the unrealistic expectations raised by the ideals of beauty conveyed by Western media and deconstructs the myths of this ideal. Although the participants increased appreciation of their bodies, the level of WBI did not significantly decrease in the short-term intervention (i.e., 1 hour per week for 4 weeks). However, Olson et al. (2018) emphasized that improved body image and satisfaction could act as a protective factor against the adverse effects of weight stigmatization. They also added that the duration of the intervention was too short to see a beneficial effect on the WBI. The other study proposed the Expand Your Horizon program, which focuses on the appreciation of body functionalities (Davies et al., 2021). Through writing exercises, participants learned to be grateful for what their body enables them to do in functional terms (e.g., stand up, walk, digest, etc.). The level of internalization of weight bias decreased significantly between the beginning and end of the program. In addition, this new appreciation of their bodies also helped to reduce the stress associated with healthcare and strengthen their ability to manage their emotions. As a result, participants felt better able to defend their needs. The authors stressed that this was a short-term study, so it is not possible to know whether the benefits observed were sustained over time. The study by Scagliusi et al. (2020) proposed a qualitative analysis examining body appreciation and responses to weight stigma following the Health at Every Size (HAES) program. Participants appreciated the intensive version of the HAES program (I-HAES) because it enabled them to deconstruct social norms of beauty and to encourage inner changes. In addition, they reported greater self-acceptance and willingly engaged in previously avoided situations (e.g., gym, public transport, intimate relationships, etc.). The participants applied the skills learned during the intervention to everyday situations (e.g., by challenging themselves in sport). In the I-HAES group, the participants gave less importance and credibility to people who expressed negative stereotypes, enabling them to be less vulnerable in these situations. In addition, they acquired the ability to deconstruct normative discourse coming from others and to choose this norm as being unsuitable. The participants were no longer afraid of the subject of food, thus encouraging the development of healthy behaviors and stress-free eating. Overall, the participants who took part in the I-HAES program gave a more positive meaning to their bodies and felt able to fight weight stigma.

Maladaptative coping has also emerged as a relevant determinant. The Transform Your Life (TYL) program (Carels et al., 2014) focused on developing and sustaining salutogenic habits, disengaging from nonhealthy behaviors, and modifying the participants’ environments. More specifically, the aim was to reduce obesogenic cues and promote cues that would maintain motivation to achieve the goals they had set. Once an environment has been reorganized to encourage an automatic response to the pursuit of a goal (i.e., weight loss), fewer self-regulatory resources (self-monitoring) are required to achieve this goal. In addition, the participants learned to identify the steps leading to salutogenic behaviors (e.g., snacking) and then to stop these behaviors. This process led to a reduction in the level of internalization of weight bias and an improvement in body image. In contrast to the participants of the NP program discussed above, participants in the TYL group continued to lose weight during the 6 months following the end of the program. Carels et al. (2014) pointed out that the support, accountability, and weekly attention provided by the competent staff delivering the interventions may have been factors in their success.

The KG-Free intervention (Palmeira et al., 2017, 2019) specifically targets weight-related avoidance experiences (WREA), which refer to the refusal to stay in touch with difficult weight- and eating-related internal experiences (e.g., craving, weight self-stigma) (Lillis et al., 2009). This intervention integrates the development of mindfulness skills, self-compassion, acceptance, and commitment. In the study, the KG-Free intervention reduced the level of WBI and increased the participants’ quality of life in relation to obesity. According to the mediation model tested, this improvement was due to a reduction in the level of self-stigmatization (Palmeira et al., 2019). However, this reduction was itself mediated by the influence of the intervention on the WREA, the feelings of shame and self-criticism, and the capacity for mindfulness and self-compassion acquired during the program. In terms of behavior, the participants reported taking part in physical activity more often and tended not to devalue themselves and to avoid uncomfortable or difficult situations less.

### Indirect effects of interventions on weight stigma outcomes

Ten of the 24 studies investigated the effect of an intervention that did not specifically target the deleterious effects of weight stigma (Berman et al., 2016, 2022; Carels et al., 2009, 2010, 2017, 2019; Haley et al., 2022; Levin et al., 2021; Mensinger et al., 2016; Wallin et al., 2018). More specifically, in these studies, research did not focus on weight stigma as the primary outcome. Of these ten studies, six primarily assessed the feasibility, acceptability, and effectiveness of their intervention and explored its effects on the level of weight-related stigma. Four of these studies examined the reduction of weight self-stigma as a moderator of the effects of their intervention.

Four of these studies offered their participants a self-help manual (Carels et al., 2009, 2017, 2019; Wallin et al., 2018). Among these four, two studies incorporated CBT techniques such as acceptance commitment therapy (ACT), which reduced the participants’ level of self-stigmatization (Carels et al., 2019; Wallin et al., 2018). However, this improvement seems to be linked to individual predispositions. Indeed, in Carels et al.’s (2009, 2017, 2019) work, the protocols used proceeded by phase. Patients had to achieve a weight-loss goal, and depending on whether they succeeded or failed, they were guided differently. If weight loss was achieved, patients continued with a self-help booklet; otherwise, group sessions were prescribed. Patients who achieved their weight-loss goal in the first phase were those who benefited most from the booklets. The other two studies (Carels et al., 2009, 2017) offered their participants a more pragmatic booklet that included tools such as calorie counting, a physical activity report, and a diet. First, Carels et al. (2009) analysed the effect on implicit and explicit weight bias. The level of explicit bias remained stable between the start and end of the program. When participants attributed more positive traits to people living with obesity (e.g., sporty, intelligent, dynamic), weight loss seemed to be greater, as did calorie monitoring. They observed no effect on implicit bias. Weight bias influences behavior and the success of the program, but the mechanisms involved are not clearly known. In another study, Carels et al. (2019) identified two individual success factors for weight loss: health literacy and calorie monitoring. Participants with higher health literacy were those who benefited most from the proposed program. The authors did not find any effect of the internalization of weight stigma on weight loss or changes in behavior.

Three of the ten offered group sessions (Carels et al., 2010; Haley et al., 2022; Levin et al., 2021). Among them, one study examined the relationship between explicit and implicit bias and the internalization of stereotypes (Carels et al., 2010). Participants followed two different programs, one focusing on lifestyle change and the other on environmental modification (i.e., reducing obesogenic cues). WBI did not seem to be associated with the participants’ level of implicit bias. Both groups saw their level of WBI decrease, and this decrease was linked to a better evaluation of body appearance. In short, after following these programs, participants attributed more positive traits to people with obesity and were less likely to internalize weight bias. However, it is important to note that the authors did not differentiate between the results of the two groups. The other two studies proposed group sessions integrating workshops centered on acceptance and commitment therapy (Levin et al., 2021) and self-compassion (Haley et al., 2022). Levin et al. (2021) offered participants an online ACT coupled with telephone coaching. This program reduced the level of IWS, reduced maladaptive behavior, and encouraged salutogenic behavior. In their pilot study, Haley et al. (2022) sought to determine whether a program to improve general self-compassion could have an effect on WBI. This program focused on applying self-compassion skills to different areas of life. They did not observe any effect on WBI. However, participants had a better appreciation of their bodies and better control over their diet following the program.

The remaining three of the ten studies combined provision of a self-help manual and group sessions (Berman et al., 2016, 2022; Mensinger et al., 2016). Berman et al. (2016, 2022) proposed the “Accept Yourself!” program, which aimed to improve physical health without necessarily prioritizing weight loss. A significant interaction indicated that participants improved their overall quality of life, decreased their weight, and WBI scores during the program. However, these effects were not maintained 1 year after the end of the program. Mensinger et al. (2016) compared a weight-neutral HUGS program (i.e., Health-focused, Understanding lifestyle, Group supported, and Self-esteem building) with a conventional weight-loss LEARN program (i.e., Lifestyle, Exercise, Attitudes, Relationships, and Nutrition). The weight-neutral program shifted the focus away from the weight-loss goal and focused more on well-being and self-care regardless of fitness (Tylka et al., 2014). Both groups experienced a decrease in the level of WBI during the program and maintained 24 months later. Participants with lower levels of WBI appear to have benefited the most from the neutral program, while those with higher levels of WBI benefited the least, regardless of the course followed. However, the frequency of use of the booklet and, more generally, of the skills acquired was not reported in this study.

## Discussion

The objective of this review, conducted in accordance with the PRISMA guidelines, was first to identify the various forms of intervention that address the consequences of weight stigma on obesity management. Then, we sought to identify the psychological (e.g., self-esteem, body image, etc.) and behavioral benefits (e.g., development of healthy behaviors). Some interventions focused more on the participants’ environments and skills, while others more specifically addressed the issue of weight stigma with their participants.

The studies included in this review were selected based on their methodological robustness, diversity of approaches, and focus on psychosocial determinants such as WBI, body image, or self-esteem. All studies employed evidence-based frameworks like CBT, ACT, or self-compassion, demonstrating their alignment with review’s objectives. They explicitly addressed weight stigma or its psychological correlates, such as emotional regulation, ensuring direct applicability to understanding broader impact of weight stigma on obesity management. The diversity of formats, ranging from self-help manuals to group-based programs, provided valuable insights into the advantages and limitations of various intervention types.

Weight stigma affects outcomes in obesity management through several mechanisms. It exacerbates stress, emotional distress, often leading to maladaptative behaviors such emotional eating or avoidance of healthcare. Studies included in this review demonstrated that intervention focusing solely on reducing weight stigma often achieved limited long-term improvement in quality of life. This suggests that weight stigma should be considered within a broader framework, alongside factors like body image, coping strategies and self-esteem, which influence individuals’ perception of and vulnerability to weight stigma. Thus, interventions incorporated these additional factors observed more robust and sustained effects. Secondly, the duration of the intervention was identified as an important characteristic. Longer-term interventions led to greater benefits, which were generally maintained after the end of the intervention. Several factors may explain this effect. Firstly, the participants were able to get together on a regular basis, thus creating a bond and group cohesion. Secondly, the longer duration would have enabled participants to better understand and assimilate the content of the intervention, notably through the feedback provided by the researchers. In addition, some studies included coaching during the intervention, helping to keep participants motivated and involved. Finally, the tools used were also a relevant feature that emerged from the interventions analyzed. Two formats were favoured, self-help manuals or group sessions, and some studies combined the two. Self-help manuals provided participants with a permanent source of support. These manuals could incorporate CBT techniques or advice on how to modify one’s environment and promote salutogenic behaviors. Group sessions also incorporated CBT techniques, and had the advantage of maintaining group cohesion and thus fostering social bonding.

All the studies in this review incorporated CBT and psychoeducational techniques to help participants better manage their condition navigate challenging situations. Many intervention included a self-help manual, providing practical tools for managing obesity. These manuals proved effective in improving body image, regulating food-related emotions, and reducing WBI. However, their success depends on participants’ ability to fully understand and utilize the content. Thus, greater health literacy is a key factor in ensuring the success of self-help manuals (Carels et al., 2017).

This review identified WBI as a common variable across nearly all these studies, playing a pivotal role in shaping patients’ perceptions of and responses to weight stigma. Intervention that reduced WBI led to improvement in body image, self-esteem, depressive symptoms, and other psychosocial determinants (Pearl et al., 2020a,b). However, addressing WBI aline is insufficient to mitigate its harmful effects fully. It is equally important to target related factors, particularly self-stigmatization variables (e.g., body image, self-esteem, feelings of shame, etc.). For instance, emphasizing body functionality rather than appearance has been shown helping to reduce healthcare-related stress and WBI levels (Davies et al., 2021). While these studies establish a link between WBI and these determinants, the underlying processes remain unclear. Only three studies (Palmeira et al., 2017, 2019; Potts et al., 2022) investigate the mechanisms mediating the reduction in internalized weight bias. Findings suggest that a decrease in WBI does not directly does not directly improve quality of life but acts through other variables (e.g., self-deprecation) (Palmeira et al., 2019).

Although these studies all tend to confirm the benefits of an intervention on the various psychosocial determinants, they have several limitations. The most common limitation is the small sample size and homogeneity (i.e., predominantly Caucasian women). In addition, most of these studies highlight the benefits of their interventions, but only three of them analyse the processes by which these changes take place (Palmeira et al., 2017, 2019; Potts et al., 2022). The qualitative dimension is also little explored, even though it could provide information on participants’ experiences and perceptions of the intervention. From a methodological perspective, potential biases inherent to the PRISMA review process must also be acknowledged. The search strategy was limited to three major databases (PubMed, APA PsycArticles/PsycInfo, and Web of Science), which may have excluded relevant studies indexed in other sources. Only English-language, peer-reviewed articles were included, potentially omitting significant non-English research and grey literature. Moreover, the inclusion criteria focused on studies published between 1975 and 2024, which might have excluded older findings.

In a society where stereotypes of obesity can be pervasive, it is vital to put in place measures aimed at strengthening the resources of people living with obesity. It seems necessary and important to offer a comprehensive program incorporating both remedial techniques derived from psychology (e.g., ACT, self-compassion, self-affirmation) and more pragmatic exercises such as a diet diary. In addition, the environment in which the program takes place must be supportive and provide feedback to participants in order to maintain their motivation and encourage their progress (Levin et al., 2018; Potts et al., 2022). It is also important for patients themselves to be involved in making changes to their environment and lifestyle (Carels et al., 2014) in order to derive lasting benefits over time. Although short-term interventions can lead to an improvement in quality of life, this improvement is not always maintained and is not of great magnitude (Forbes et al., 2020; Lillis et al., 2009). Thus, the duration of the intervention is, an important variable to consider, as it is necessary to have sufficient time to assimilate the content of a program and learn how to apply it in other areas.

Future research will, therefore, have to identify the nature of the link between the internalization of weight bias and the psychosocial determinants contributing to improved quality of life. We have identified at least three priority focus areas for research.

### Research focus 1: to establish the relationship between reduced internalization of weight bias and improved quality of life

We assume that the various stigmatizing contexts can have deleterious effects on the quality of life of people suffering from obesity. These contexts contribute to aggravating the condition because individuals can find themselves powerless and without solutions. Remedying the deleterious effects of weight stigma could, as a consequence, have a positive effect on the quality of life of this population. Therapeutic patient education (TPE) seems to be a relevant approach because it focuses on identifying the needs and skills of the individual. It places patients at the heart of care and works with them to develop their health skills so that they can live better with their chronic illness. This type of program does not focus directly on the usual concerns such as weight loss and prescribing a specific diet but rather on developing and strengthening useful healthcare resources. To our knowledge, no TPE program integrates psychosocial aspects such as weight stigma. We would hope that participation in such a program would improve quality of life and empowerment (i.e., a sense of self-efficacy).

This hypothesis could be tested with a population of people with obesity integrated into a care pathway in a hospital center using two methods: (1) a standard therapeutic education program on obesity, the content of which focuses on a better understanding of this chronic disease; and (2) a therapeutic education program on obesity enhanced by workshops and tools related to weight stigma.

### Research focus 2: examining change processes by analyzing the mediating effects of psychosocial factors on the internalization of weight bias

The second research axis will focus on identifying the psychosocial factors responsible for change and the benefits perceived by patients. We assume that working solely on stigma issues is not enough to improve quality of life. Patients’ vulnerability to stigmatizing contexts may be influenced by other factors such as self-esteem, body image, or coping strategies. For example, greater acceptance of one’s own body size, combined with improved self-esteem, may lead individuals to be less preoccupied with the gaze and judgment of others (Scagliusi et al., 2020). These psychosocial factors could, therefore, be potential mediators of perceived benefits and should be tested through statistical mediation analyses. We will try to identify the processes by which reducing the level of internalization of weight bias can improve the quality of life of individuals living with obesity.

### Research focus 3: qualitative analysis of the experience and perceived benefits of following a program focusing on weight stigma

Finally, in this last line of research, it will be necessary to understand how patients benefit from the intervention and to what extent they apply the skills they have acquired to everyday situations. Quantitative measures provide a better understanding of how psychosocial factors influence each other but do not capture the entire process of change. In order to explore this area, it will be necessary to carry out recorded interviews with patients taking part in TPE programs. Analysis of the content of these interviews will help to flesh out the quantitative results obtained but also to better understand the possibility of a lack of benefits.

## Conclusion

The present review underscores the importance of tailored interventions to address the multifaceted aspects of weight stigma and its impact on individuals’ well-being. Although weight stigma management appears to be linked to the quality of life of people with obesity, the mechanisms of action involved remain unclear. Thus, it is necessary to study the involvement of psychosocial factors (e.g., self-esteem, body image, etc.) in this relationship.

## Data Availability

The original contributions presented in the study are included in the article/supplementary material, further inquiries can be directed to the corresponding author.
